# Supplementary material for: Identification and functional analysis of non-coding regulatory small RNA FenSr3 in Bacillus amyloliquefaciens LPB-18
Source: PeerJ. 2023 May 15;11:e15236. doi: 10.7717/peerj.15236 (PMC10194069; doi:10.7717/peerj.15236)
Supplement: Supplemental Information 4 [file peerj-11-15236-s004.zip › KO/CK-vs-T1_map/map04976.html]

KEGG PATHWAY: Bile secretion - Reference pathway


|  |  |
| --- | --- |
| **Bile secretion - Reference pathway** |  |

[
Pathway menu
| Organism menu
| Pathway entry
| Show description
| User data mapping
]

Bile is a vital secretion, essential for digestion and absorption of fats and fat-soluble vitamins in the small intestine. Moreover, bile is an important route of elimination for excess cholesterol and many waste product, bilirubin, drugs and toxic compounds. Bile secretion depends on the function of membrane transport systems in hepatocytes and cholangiocytes and on the structural and functional integrity of the biliary tree. The hepatocytes generate the so-called primary bile in their canaliculi. Cholangiocytes modify the canalicular bile by secretory and reabsorptive processes as bile passes through the bile ducts. The main solutes in bile are bile acids, which stimulate bile secretion osmotically, as well as facilitate the intestinal absorption of dietary lipids by their detergent properties. Bile acids are also important signalling molecules. Through the activation of nuclear receptors, they regulate their own synthesis and transport rates.

|  |  |  |
| --- | --- | --- |
| Reference pathway | 184% 150% 122% 100% 82% 67% 55% | 图片下载 |
